# Strengthening and measuring research impact in global health: lessons from applying the FAIT framework

**DOI:** 10.1186/s12961-019-0451-0

**Published:** 2019-05-06

**Authors:** Rebecca Dodd, Shanthi Ramanathan, Blake Angell, David Peiris, Rohina Joshi, Andrew Searles, Jacqui Webster

**Affiliations:** 1 The George Institute for Global Health, University of New South Wales, 1 King Street, Newtown, Sydney 2042 Australia; 2 Hunter Medical Research Institute, Locked Bag 1000, New Lambton, NSW 2305 Australia; 3 School of Medicine and Public Health, University of Newcastle, University Drive, Callaghan, NSW 2308 Australia

**Keywords:** Research impact, Translation, Economic impact, Low-income countries

## Abstract

**Background:**

To date, efforts to measure impact have largely focused on health research in high-income countries, reflecting where the majority of health research funding is spent. Nevertheless, there is a growing body of health and medical research being undertaken in low- and middle-income countries (LMICs), supported by both development aid and established research funders. The Framework to Assess the Impact of Translational health research (FAIT) combines three approaches to measuring research impact (Payback, economic assessment and case study narrative). Its aim is to strengthen the focus on translation and impact measurement in health research. FAIT has been used by several Australian research initiatives; however, it has not been used in LMICs. Our aim was to apply FAIT in an LMIC context and evaluate its utility.

**Methods:**

We retrospectively applied all three FAIT methods to two LMIC studies using available data, supplemented with group discussion and further economic analyses. Results were presented in a scorecard format.

**Results:**

FAIT helped clarify pathways of impact for the projects and provided new knowledge on areas of impact in several domains, including capacity-building for research, policy development and economic impact. However, there were constraints, particularly associated with calculating the return on investment in the LMIC context. The case study narrative provided a layperson’s summary of the research that helped to explain outcomes and succinctly communicate lessons learnt.

**Conclusion:**

Use of FAIT to assess the impact of LMIC research was both feasible and useful. We make recommendations related to prospective use, identification of metrics to support use of the Payback framework, and simplification of the economic assessment, which may facilitate further application in LMIC environments.

**Electronic supplementary material:**

The online version of this article (10.1186/s12961-019-0451-0) contains supplementary material, which is available to authorized users.

## Background

There is a growing interest among both research funders [1–3] and academics [4–7] in identifying and measuring the social, environmental and economic benefits of research. Calls to better describe ‘impact’ are driven by the need to improve accountability, ensure relevance and inform funding [7–9]. Whether research outcomes can be ‘translated’ or applied in the real world is seen as one important way of assessing benefit [4, 5, 10]. Further, identifying pathways to impact during the design of research programmes can improve the quality and integrity of research by clarifying purpose and end-users [10, 11]. Interest in the benefits of health and biomedical research has been prominent in broader discussions on research impact [7, 9] due to the large amount of public funding it attracts [5] and the importance of tailoring outputs to the needs of clinicians and patients [5, 12].

Calls for evidence of impact have in turn catalysed work on how to measure it, and a range of approaches have been developed globally [8, 12–14]. One of the earliest and most widely-used [8, 9] approaches is the Payback model, introduced by Buxton and Hanney [15]. It aims to capture benefits in a range of areas such as knowledge generation, health services improvement and policy development, and has been adapted or modified a number of times [16]. Economic assessment (i.e. monetising research impacts) is also widely used, though typically at high levels, for example, aggregating research benefit nationally or in specific programmes over decades [17–19]. Project-specific approaches to measuring economic impact are emerging [8], though they have been critiqued for over-reliance on modelling and questionable assumptions [7, 17]. Narratives are a third, validated approach to describing impact, providing a summary of the research process and outcomes and have been the basis of the Research Evaluation Framework in the United Kingdom. Narratives have the advantage of being able to explain the complex (and often multi-directional) process through which impact occurs [7, 10, 11].

To date, efforts to measure impact have largely focussed on health research in high-income countries (HICs) [7, 8], reflecting where the majority of health research funding is spent as well as the limited infrastructure and capacity for health research in low- and middle-income countries (LMICs) [20, 21]. In 1990, the Commission on Health Research for Development, a consortium of global health agencies, researchers and development partners, identified the ‘90/10 gap’, i.e. that less than 10% of global health research spending is devoted to diseases or conditions that account for 90% of the global disease burden. This led to calls for a more equitable and systematic approach to prioritising health research investments [22, 23] and for development partners to devote 5% of official development assistance (aid) for health to research, as well as for LMICs to increase their own health research spending [24].

These calls have in part been answered – there is now more research in and on the health needs of low-income countries [25, 26] and a growing number of development partners are active in health research [27], including the United Kingdom’s Department for International Development, the United States Agency for International Development, and the Bill and Melinda Gates Foundation; these three agencies acknowledge the need to monitor the impact of their research investment [28–30]. Though none have standalone research impact frameworks, their policy documents refer to the need for research investments to create new knowledge and inform decision-making [28], build capacity for research in LMICs [28, 29] and facilitate local adaptation of evidence-based approaches (e.g. through implementation research) [29, 30] – all common elements of the ‘Payback’ model. Similarly, major funders of domestic health research in the United Kingdom, the United States and Canada now also fund research in LMICs directly [31–34] and through international collaborations [35]. These agencies do not appear to have standalone impact frameworks specific to their international collaborations (the United Kingdom Medical Research Council uses the Department for International Development framework); however, their domestic research impact models highlight knowledge generation and influence on policy and practice [36], economic growth (measured through links with business) and long-term health and environmental impact [2].

Thus, despite vast differences in health needs and research capacities in HIC and LMICs, there are similar expectations of what health research should achieve and how its impact should be measured in both contexts. As research investment in LMIC environments continues to grow, a better understanding of the challenges associated with research translation and measuring impact in LMIC contexts is likely to be useful. This study applies a research impact framework developed in a HIC (Australia) to research carried out in the Pacific and Indonesia. Our aim was to evaluate applicability, identify strengths and weaknesses, and make recommendations to support further use.

## Methods

We carried out a rapid search for health research impact frameworks and selected the Hunter Medical Research Institute’s (HMRI) Framework to Assess the Impact from Translational health research (FAIT). Based on an extensive review of existing impact frameworks and with input from potential users, FAIT combines the three most commonly used approaches to impact assessment [10]. The first, based on the ‘Payback’ model, identifies ‘domains of benefit’. While each domain of FAIT is based on an existing approach to research impact, the combination of these approaches into a single tool is novel. Domains can be adapted to the research project under review but suggestions proposed by FAIT include knowledge generation, impacts on policy, clinical practice, health services or population health, and economic benefits. The second method comprises a cost–benefit analysis that compares costs (of the research itself and of implementing research recommendations), to social, environmental and economic benefits (expressed in monetary terms) that flow from implementation. Again, categories of benefit are flexible and left to the discretion of those completing the assessment. The third section is a short narrative that provides a summary of *“how translation occurred and how research impact was generated”* [10]. The text is structured around common sub-headings (need, research response, outcome, impact, lessons) and its purpose is to contextualise quantitative findings and explain outcomes.

We selected FAIT for three reasons. First, its mixed-methods approach, combining the three main approaches to measuring research impact provided an opportunity to test a range of impact measurement approaches in the LMIC context. Second, the framework emphasises translational health research, and is therefore well suited to the research projects we sought to review, which aimed to influence policy and practice. Third, FAIT can be applied to a range of research methods, from qualitative studies to implementation research to clinical trials, and so has a potential for wide application. FAIT is currently being applied to five projects within an Australian Centre for Research Excellence [37] but has not yet been applied in an LMIC context. HMRI colleagues agreed to engage in our study, adding value by reviewing our use of the FAIT tool.

Between March and September 2018, we applied FAIT to two recently completed research projects, namely (1) a programme to reduce salt consumption in two Pacific Island countries, Samoa and Fiji, and (2) the introduction of a digital health tool, SMART*health,* to improve the quality of diagnosis and treatment of cardiovascular diseases in East Java, Indonesia.

We chose these projects because good documentation was available from which data could be extracted. For the Pacific Salt project, there was a study protocol, process evaluations, impact evaluations and intervention costings for each Pacific country [38, 39]. For SMART*health,* an end-of-project completion report had been prepared for the funder, which included clinical results and a cost-effectiveness analysis. While FAIT is designed to be applied prospectively to encourage research translation and ensure the required evidence of impact is collected along the way, retrospective application represented a feasible approach to determine the applicability of FAIT in the LMIC context.

Our study was carried out in two stages. In stage one, we completed a first draft of the impact framework (presented in the FAIT scorecard format). We drew on existing documents to source the majority of data required, supplementing this with additional discussion and data mining where needed. The process was led by RD, who was not part of the research teams responsible for the two chosen projects, with support from BA to complete the economic analysis. The leads of each research team provided relevant documents and reviewed and amended RD’s first draft. In stage two, the lead author of the FAIT framework (AS) and the person leading its application and translation (SR) provided feedback and comment, which was critical to the process of refinement, including identifying additional areas of impact. While previous applications of FAIT have focussed on gathering impact data for the project under review, this study also considered the framework itself, and its applicability to a developing country context.

## Results

Tables 1 and 2 present populated impact scorecards for the Pacific Salt project and the SMART*health* project. We found application of the ‘domains of benefit’ section to be feasible and useful – it helped to generate evidence of impact, including new data, in a range of areas not documented in existing project evaluations. For the Pacific Salt project, this included impacts on knowledge advancement and capacity-building, as well as indirect, positive impacts on the Samoan and Fijian economies through generating employment (in the research team) and spending of project funds (Table 1). For example, in relation to policy development, use of the framework drew attention to networks established with policy-makers, and the learning generated on the political economy of working with the food industry in Fiji, which research project leaders were aware of but had not previously documented. Support and prompting from HMRI was critical to the identification of these domains, and to the process of describing and quantifying specific benefits within them.

**Table 1:** Impact scorecard for the Pacific Salt project intervention, Fiji and Samoa, Pacific

		Metrics	Outputs/outcomes
Domains of Benefit	Advance knowledge	• Peer-reviewed publications • New datasets established	• 14 papers in total: 7 papers resulting directly from the research programme (1 protocol, 2 baseline, 2 impact, 2 process evaluation) and 7 papers linked to the project (2 systematic reviews, 1 methods paper on developing targets, and 4 cross-cutting papers in collaboration with other researchers) • Presentation at the World Congress on Public Health in Melbourne in 2017 and the Australian Food Governance Conference in 2016 and workshops for staff at the George Institute and University of New South Wales • 2 new national datasets established, including baselines on population salt levels for two Pacific countries where no previous data available
	Capacity-building	• Academic qualifications • Knowledge and capabilities of health workforce	• 1 PhD (Sydney) and 2 Masters (1 Sydney and 1 Fiji) qualifications • 4 authors from Fiji and Samoa included on 7 academic publications, including 1 led by a Fijian author • 6 salt reduction training sessions for health staff, Ministry of Health managers, health volunteers • 4 research assistants trained in data collection related to salt monitoring in each country • 3 project staff from Fiji and Samoa attended training on implementation science • 14 Samoan staff attend data-analysis training in Samoa
	Healthy eating: education, behaviour change and healthy food environments	• Consumer knowledge and awareness of health risk associated with salt • Salt intake (g/day) • Hospital’s food environment	• 9% increase in population understanding of adverse effects of salt in Samoa • 16% reduction in population that always/often add salt to foods in Samoa • 28% increase in population reporting using spices instead of salt during cooking in Samoa • 70% of people in both countries aware of the salt reduction campaign and reported having seen promotional materials • 1 hospital (Fiji) removes salt shakers from staff dining room, and salt content of hospital meals reduced • No salt shakers on tables introduced into Fiji’s restaurant grading scheme but no data on compliance
	Engagement and networking	• Public–private dialogue on food policy	• 2 forums and 10 face-to-face meetings with individual companies in Fiji each year • Multi-sectoral working groups (Agriculture, Trade and Commerce, Education, Finance, Women and Culture) convened to oversee programme implementation in Fiji and Samoa
	Economic impact	• Reduced health system costs • Current and future income of staff associated with study • New research financing	*Unable to calculate due to lack of data but could be done using the following approach:* • Model economic value of reduced salt intake based on reduced burden of hypertension and associated decrease in health system costs • Additional lifetime income of 2 PhD students (vs. Masters) and 1 Masters (vs. Bachelor) • New grant of AUD$ 150,000 secured
Social Return on Investment	Cost of research	Total project cost (a)	AUD$ 1.2 million
	Cost of using research outcomes	Based on cost of the interventions trialled in the research (b)	Total cost of salt reduction campaign implemented during project: AUD$ 177,000 or AUD$ 0.19 per capita Scaled up campaign costed at approximately AUD$ 500,000 per year per country (2.5 million over 5 years per country)
	Benefit converted AUD$ values	Change in salt consumption converted to DALYs saved over life of cohort (c)	*Could be completed using the following approach:* Economic modelling based on low, medium and high reductions in salt intake, which should occur if health promotion activities modelled on those piloted in this study are delivered, and if legislative and regulatory changes are implemented. This would provide an estimate of avoided morbidity over a specified period, and from this health systems costs can be estimated
	Social return on investment	c / a + b	*Not possible due to lack of data*
Case study	Need*:* Approximately 40% of the adult population in Samoa and 31% in Fiji have high blood pressure (BP). Excess salt intake is known to be a key contributor to raised BP, which is in turn a major risk factor for cardiovascular disease (CVD). No accurate information was previously available on salt intake in either country. Further, while reducing salt intake is a known cost-effective intervention to prevent CVD, very little is known on how to achieve this in low- and middle-income countries (LMICs) and no Pacific country had run a successful salt reduction campaign. Research response: Researchers collected the first baseline survey on salt consumption in each country and used this to design a multi-pronged salt reduction campaign to increase consumer awareness of health risks of salt, and reduce salt content in processed foods through engaging food manufacturers. Educational materials, including pamphlets, posters, DVDs and presentations, were produced for each country. In Samoa, the research team worked with Government to amend the Food Act to introduce labelling of salt content and introduce mandatory salt targets. A rigorous evaluation process was carried, providing the first ever evaluation of a national salt reduction campaign in a LMIC. Outcome: The project delivered new knowledge on salt levels in two Pacific countries and showed (for the first time) that it is possible to achieve some level of behaviour change in a relatively short timeframe and at low cost in a Pacific context. Consultations between food industry and Government were the first public–private consultation on salt and health in these countries. Training and capacity-building outcomes were extensive, and likely to yield an economic return in Australia and the Pacific through improved employability. Academic networks between Australia and the Pacific were established and new funding secured. Impact: The health promotion campaign had high penetration in both countries leading to significant improvements in consumer knowledge and some changes in behaviours. In Fiji, the new Food and Health strategy (2018–2022) commits to reduce salt, fat and sugar intakes. Health workers – a key source of information for the public on healthy eating – demonstrated increased knowledge of health risks of salt. Pacific specific guidelines on measuring salt intake have been produced, endorsed by WHO, and lessons from this project contributed to WHO’s “SHAKE” guidance on salt reduction. Lessons: Three years is too short to demonstrate a reduction in mean population salt intake; any future campaigns would need to expand coverage and run for longer. Further, high consumption of processed food in the Pacific suggests that changes to discretionary salt intake will be insufficient – broader changes to the food environment will require strong government engagement since food industry stakeholders are unwilling to change practice voluntarily, preferring to wait for clear direction from Government.

**Table 2:** Impact scorecard for SMART*health* intervention, Malang, East Java, Indonesia

		Metrics	Outputs/outcomes
Domains of Benefit	Advance knowledge	• Peer-reviewed papers • Dissemination of project findings • Tailored decision support tools	• 2 peer-reviewed publications under review • Plain language reports written and discussed with Malang District Health Authority and Indonesia’s national health insurance agency • 1 new clinical decision support algorithm for cardiovascular disease (CVD) screening and treatment, calibrated to Indonesian burden of disease.
	Capacity-building and networking	• New research networks	• 5 staff from University of Brawijaya are co-authors on peer-reviewed papers • 4 University of Brawijaya staff and 1 Malang district health official are co-applicant on two follow-up grants
	Health systems strengthening	• Medication supply • Workforce capacity • Management of CVD services	• Stock of medication increased by 50% in four primary healthcare facilities participating in the project; medicines procured using local drug procurement system • 42 lay health workers (*Kaders*), 14 nurses and 5 doctors trained on risk factors for NCDs, proper management of high-risk CVD and use of the SMART*health* platform • A new electronic record created for every patient screened, along with a system to share records between *Kaders* and doctors (working at a different facility), previously none existed
	Health outcomes	• Access to CVD care in the community • Quality of care	• *Kaders* screened 11,000 people (91% of target cohort) • 22% of those screened found to be at high-risk of a CVD event, of these 100% referred to a primary healthcare facility • 91% of those referred followed up by *Kaders* at least once • 80% of high-risk patients got appropriate medication, compared to 16% in the control population, resulting in a difference in mean systolic blood pressure of 13 mmHg between high-risk individuals in intervention and control villages • Modelling suggests there will be 59 fewer CVD events in the intervention villages compared to control population over 5 years; if expanded nationally, 73,000 CVD events could be averted over 5 years
	Economic impact	• Health system savings • Research team income • New financing	• The research team wages contributed to the local economy • To date, one follow-up grant of US$ 70,000 secured • 6 staff employed by the project for 12 months received total wages of 266,953,848 Rupiah • To date, 1 grant secured (~US$ 700,000)
Social Return on Investment	Cost of research	Total project cost (a)	US$ 1 million over 12 months
	Cost of using research	Cost of deploying the interventions trialled (b)	Scaling up the intervention nationally, including primary care, and pharmaceutical costs estimated at US$ 328.3 million over 5 years
	Benefit converted US$ values	Economic benefit of deploying the intervention at scale (c)	• Assuming one hospitalisation per CVD event, the intervention would save US$ 333 million over 5 years, nationally • Estimated productivity gains are US$ 192.4–384.8 million based on 6–12 months average income for those avoiding hospital • Indirect benefits of avoiding CVD events are estimated at US$ 192.4 million, based on published data
	Social return on investment	c / a + b	2.19 under conservative assumptions 2.77 under base case assumptions
Case study	Need: CVDs are the leading cause of death globally, with coronary heart disease and stroke accounting for one-third of mortality worldwide. In Indonesia at least two-thirds of those with, or at high-risk of developing, a CVD do not receive appropriate treatment and there are 470,000 coronary heart disease deaths annually. Research response: TGI’s Systematic Medical Appraisal, Referral and Treatment (SMART*health*) is a primary care intervention to support the prevention and management of common non-communicable diseases. Deployed successfully in Australia, India and China, the project SMART*health* was delivered in four villages in Malang district, East Java, Indonesia, over 12-months from April 2017. Lay workers (*Kaders*) were trained to use a mobile tablet device loaded with a clinical decision support system (CDSS) and given a blood pressure monitor and glucometer. Doctors received a different tablet, loaded with a physician-specific CDSS. *Kaders* screened adults in their community guided by the CDSS, identified those at high risk and referred them to doctors, who in turn used their CDSS to prescribe appropriate medication. *Kaders* then followed-up on high-risk patients, promoting lifestyle change and adherence to medication. Research outcome: The project delivered the first comprehensive assessment of CVD burden and access to care in any part of rural Indonesia. A high proportion of the population was screened, high-risk cases referred for treatment, and the majority followed up once back in the community. Complementary health systems support improved medicines supply and the skills of the health workforce. The project received awards from the Malang District Government and the Ministry of Health, Indonesia. Research impact: Use of recommended medications among people at high-risk was much higher in the intervention villages resulting in significant reductions in blood pressure. A key success factor was delivery of care direct to households. The intervention also enhanced the status of the female *Kaders* within their communities, improving their motivation. However, there was limited evidence of lifestyle change. Lessons: A detailed health systems assessment was critical to provide context for the intervention. This identified support needs in staff training, medicines supply and information systems that support sustainability.		

For the SMART*health* project, use of the ‘domains of benefit’ section helped to identify previously unrecognised areas of impact on knowledge generation and capacity-building of in-country partners. In addition, it prompted additional work to quantify recognised (but previously unreported) positive impacts on the health system; these included numbers of health workers trained, improvements to the medications supply, and better collection and sharing of patient data (Table 2). While these aspects were mentioned in the project evaluation, they had not been explicitly measured or identified as project benefits. For both projects, the narrative text provided a useful summary of the project and its impacts and provided an opportunity to reflect on lessons learnt.

We found generating data on the return on investment to be the most challenging aspect of FAIT, for a number of reasons. First, it required specialist input from a health economist (not available to all project teams). Second, data needed to model economic returns are often not easily available for the LMIC context, and in the case of the Pacific Salt project, were not collected during project implementation. This meant that areas of benefit identified retrospectively, such as the increased earning potential of staff in partner countries who gained skills through being involved in the project, could not be calculated. In Australia, standard pay scales for most professions are available and could be used to model such a benefit, but this is not the case for the majority of LMICs. Similarly, health gains and life years saved are commonly used measures of impact of interventions in HICs. However, they are more difficult to calculate in LMICs given the paucity of data.

Third, we found the broader context of poverty and health systems development had an impact on efficiency and hence economic return. For instance, SMART*health* provided support to allow a clinical task normally provided by a physician to be delivered by a lower-cost community health worker. In circumstances where physicians do provide this support, such a ‘shift’ would be cost saving. However, this was not the case in Indonesia, where the counter-factual was ‘no care’ so introducing the intervention represented a net cost to the health system, which in turn diminished the final social return on investment. Recognising these challenges, we attempted to monetarise the identified benefits of SMART*health,* drawing on the literature for both methods and estimates on which to base assumptions. Benefits of the intervention were modelled using estimates of the reduction in cardiovascular disease (CVD) events avoided as a result of the intervention. In summary, our assumptions were:

- A relative risk reduction in CVD events (ischaemic heart disease, myocardial infarction or stroke) of 0.80 for every 10 mmHg reduction in systolic blood pressure, based on a recent systematic review and meta-analysis of randomised clinical trials [40].
- One hospitalisation per CVD event.
- Disability weights for people with CVD were adopted from the Global Burden of Disease Study, using an estimated weighted average from myocardial infarction and moderate to severe stroke weights, resulting in a disability-adjusted life year (DALY) weight of 0.39. A disability weight of 1 was used to reflect the dead health state and used to calculate the years of life lost [41].
- Death rates resulting from CVD events were estimated using results from the literature for middle-income nations [42].

Using these assumptions, we then calculated:

- The reduction in CVD events resulting from the reductions in blood pressure found through the trial, projected over a 5-year period.
- The savings to the health system, based on the average cost of hospitalisations for CVD events in Indonesia.
- The health gains for the population resulting from the intervention in terms of DALYs averted. Using estimates from the literature [43], we estimated each healthy life year gained to represent productivity gains of 6–12 months of per capita Gross National Income. We believed this to be a conservative estimate, given the average age of the cohort targeted by the intervention was 59 years, average life expectancy in Indonesia is 69 years [44], unemployment is relatively low at 6.9% [45], and almost two-thirds work in the informal sector where there is no mandatory retirement age [46].
- Indirect and non-medical cost savings; using estimates from the literature, indirect benefits were valued as half the Gross National Income of Indonesia per capita per healthy life year gained as a result of the intervention.

These calculations were then compiled, divided by the cost of the research and delivering the intervention, and used to determine the estimated social return on investment (Table 2). Our approach was similar to one that might be used in a HIC environment, yet the resulting calculations were less robust given our reliance on estimates from outside Indonesia. We were unable to complete a similar set of calculations for the Fiji Salt Project due to lack of data collected during the project itself, though we did outline an approach for doing so (Table 1). Cost–benefit approaches are rarely used in LMICs, in part due to the types of constraints we encountered, including lack of a standardised approach and lack of data on which to base assumptions.

## Discussion

Good practice in the delivery of aid and development assistance, including aid for health, has long emphasised principles of local ownership, effectiveness and sustainability [47, 48]. Accordingly, many research projects designed and implemented in low-income contexts intuitively emphasise engagement of local stakeholders, use of local systems and measurement of meaningful results (beyond academic outputs), suggesting that research impact models should be a ‘natural fit’ with LMIC-based health research.

There is recognised tension between the linear approach to impact implied by impact models, and the understanding that interactions between researchers and end-users are complex and iterative [10, 11, 49–51]. Indeed, reviews in HICs suggest the use of research impact models has favoured quantitative, empirical studies that can describe a clear, unambiguous outcome [52] and where economic returns are likely to be high [9]. In LMIC contexts, weak health governance and implementation environments [53, 54] mean the role and influence of research ‘evidence’ is even more problematic, and therefore measurement of impact even more challenging. If, as HIC reviews suggest, the use of impact models is cementing an existing bias in research funding towards statistical measures [5] at the expense of experimental or qualitative research design [52], this may be detrimental to LMIC research, where research infrastructure is often lacking, and qualitative methods are particularly needed to explain the poorly understood [53] governance environment in which research occurs.

Nevertheless, our experience suggests that impact models can play a useful role in hypothesising pathways through which impact is expected to occur, which can in turn prompt consideration of which stakeholders need to be engaged, what advocacy work (alongside research) may be required, and what vested interests could act as a barrier to uptake. This may be particularly relevant in LMIC environments and points to the need to consider research impact pathways prospectively, during the design of the research process, rather that retrospectively as we have done in this study.

### Calculating economic benefit and return on investment

The challenge of valuing human life and calculating economic return on health investments is recognised in HIC contexts [8]. Our experiences suggest this challenge is exacerbated in the LMIC environment and, consequently, research projects in LMICs may struggle to show a positive return on investment. Issues include the following:

- Low wages, high-levels of informal sector employment and/or unemployment mean that productivity gains (as commonly measured, in terms of income) associated with extending healthy life are difficult to estimate.
- Poor levels of population health and low life expectancy (relative to HICs) may obscure gains in healthy life.
- Poor coverage of essential health services means that introducing a new service, however essential and cost-effective in and of itself, may represent a net cost for the system (as no service was previously provided) diminishing the level of return.
- The dearth of studies from LMICs on non-medical and indirect costs, such as transport to health facilities [55], make it difficult to estimate these, though they are often considerable [56, 57].
- Overarching all of these issues is the fundamental challenge of poor quality health data in many LMICs [58] and the likely low statistical accuracy of globally standardised measures such as the DALY in LMIC contexts, which are relied on to estimate cost savings and economic benefits [59].

Cost-effectiveness analyses whereby the value of interventions is assessed in terms of natural units (for example, cost per CVD event avoided) or cost–utility analyses that assess interventions in terms of utility gained (for example, DALYs) are more common than cost–benefit analyses (where all benefits are monetised) in the health sector, including in LMIC contexts [60]. In addition to being more straightforward to estimate, cost-effectiveness allows a consideration of the relative value of an intervention, which can be used to inform a ‘business case’ on whether or not to implement the intervention more widely; such data is especially important in LMIC contexts, where resources are often scarce.

Cost effectiveness analyses are therefore a critical component of determining the broader societal ‘return’ on research investment (as FAIT attempts to do). Nevertheless, practical challenges remain in performing a full cost–benefit analysis in LMIC contexts, as we have demonstrated. Equally, in qualitative studies where research benefits cannot be monetised, such as a change in perceptions or attitudes, a cost–consequence analysis may be more applicable.

Finally, it is worth acknowledging that, while the Payback and narrative components of FAIT consider impact retrospectively, based on empirical evidence, the social return on investment models projected economic returns into the future. This may appear an anomaly; however, it is common practice for economic analysis to contain an element of forecasting given the challenge of demonstrating economic impact within the short time frame of a research project.

### Suggestions for application of FAIT in LMICs

#### Prospective use with programme logic model

We applied the FAIT framework retrospectively, yielding important insights and new knowledge on study impacts. However, greater benefit is likely to come from applying the framework prospectively and in combination with a *‘*programme logic model’ as intended by FAIT’s authors [10]. For example, prospective application of FAIT can help ensure relevant data is captured in the monitoring frameworks. In the Fiji example, we were unable to complete a social return on investment due to lack of data – a prospective application of FAIT would have indicated these data gaps. Equally, prospective application of FAIT aids consideration of potential positive and negative programme externalities. In Malang, for example, prospective application may have highlighted the potential impact of the intervention on the workload of community health workers, and led to monitoring of any adverse impact on other health tasks they performed, e.g. in maternal and child health.

Programme logic models, also called ‘theory of change’ models, are commonly used in the design of development assistance (aid) programmes [61] to identify areas of potential impact and understand the process through which change occurs. Many LMIC studies bridge the disciplines of research and development, and are thus well suited to use of programme logic methodologies. The FAIT programme logic model, in line with models commonly used in aid programmes, identifies the need or issue to be addressed, activities, expected outputs, end-users of those outputs and anticipated impact [10]. Additional file 1 published with this paper provides two examples of the application of the FAIT modified programme logic model to current LMIC research projects, demonstrating its feasibility.

#### Menu of metrics

The ‘domains of benefit’ section is a key strength of FAIT allowing identification of a range of benefits beyond the intervention/process that is the subject of the study. This is particularly useful in an LMIC context, where the ‘process of doing research’ may itself have positive externalities, for example, related to capacity-building for research, supporting local policy-makers, building the skills of the health workforce, or the economic impact of research project spending. However, our experience suggests that users of FAIT may need guidance (prompting) to identify and capture such benefits. The initial list of potential domains provided by FAIT is helpful; however, further suggestions on possible metrics linked to each domain would be useful, for example, on specific areas of potential economic benefit and how to calculate these, or how to measure the sustainability and impact of a knowledge network established during a study. This would help to ensure appropriate data collection is built into study design (e.g. on salary scales), in turn facilitating calculation of return on investment.

#### Calculating economic returns

We found calculating cost–benefit to be challenging and the results to have weak validity given issues of data quality and reliance on assumptions. Furthermore, we believe that there are many contexts where it will not be possible or meaningful to undertake cost–benefit analyses due to lack of data.

Nevertheless, as interest in measuring research impact grows, and is inevitably applied to LMIC contexts, further research on how to approach this challenge is likely to be useful.

Where cost–benefit analysis is not possible, cost-effectiveness analysis may provide a practical alternative. Especially where the focus of research relates to an intervention or service delivery change that can be costed, cost-effectiveness analysis should be routinely done as part of the intervention evaluation process. Such data can contribute to a business case to scale up interventions trialled during research by projecting future returns and can also inform future research funding investment. More broadly, incorporating any form of economic analysis into research impact assessment provides a valuable perspective, re-emphasising the imperative to ensure all spending choices deliver value for money – particularly important in LMIC contexts. As discussed above, conducting prospective analysis is important to identify (and make arrangements to collect) data required to conduct economic analysis.

### Strengths and limitations

A key purpose of FAIT is to encourage research translation. To this end, it is designed for use throughout the implementation of research projects. We did not use the tool in this way – rather, we applied it retrospectively. Even so, we found it yielded useful findings. Further, we did not validate our impact claims through additional project evaluation as required by some impact templates [52]. However, an independent researcher led the process and HMRI’s involvement provided a level of external scrutiny. Indeed, our pragmatic approach to assessing impact was a strength of this study as it responds to a common critique of impact frameworks, namely that they take too long to complete and are too expensive to implement [11]. This approach was facilitated by the fact that the projects reviewed already included a significant focus on research translation through involvement of stakeholders (end users), and in the case of the Pacific Salt project, a comprehensive process evaluation [38, 39]. This points to a further limitation of our study, namely that we focussed exclusively on implementation research. Applying FAIT to other types of research project designs in LMICs will allow broader assumptions to be made about its applicability within the LMIC context.

## Conclusion

Though developed to measure impact in Australian health systems, FAIT can be applied to research projects in LMICs. We found the mixed-methods approach to assessing impact to be a key benefit of FAIT. While we encountered challenges calculating return on investment, the use of the FAIT framework helped illuminate data gaps and highlighted the importance of considering affordability. We make suggestions that support further applications of FAIT in LMICs and, we hope, will contribute to an emerging conversation on how best to measure research impact in LMICs. In this context, future research that tests the applicability of other high-income research frameworks in low-income environments may be useful. Capacity-building for any staff using the framework is likely to be a worthwhile investment.

## Additional file

Additional file 1: Example of application of FAIT programme logic model for *Health workforce study, India.* Example of application of FAIT programme logic model to *Intervention to reduce salt intake, Pacific. (DOCX 92 kb)*

## Abbreviations

CVD: cardiovascular disease
DALY: disability-adjusted life year
FAIT: Framework to Assess the Impact of Translational health research
HIC: high-income country
HMRI: Hunter Medical Research Institute
LMICs: low- and middle-income countries
