# Supplementary material for: Strengthening and measuring research impact in global health: lessons from applying the FAIT framework
Source: Health Res Policy Syst. 2019 May 6;17:48. doi: 10.1186/s12961-019-0451-0 (PMC6501392; doi:10.1186/s12961-019-0451-0)
Supplement: Supplementary file 1 — Example of application of FAIT programme logic model for Health workforce study, India. Example of application of FAIT programme logic model to Intervention to reduce salt intake, Pacific. (DOCX 92 kb) [file 12961_2019_451_MOESM1_ESM.docx]

**Additional File 1: Example of application of FAIT Program Logic Model for *Health workforce study, India***

**Issue/**

**Need**

**Aims and activities of the research**

**Outputs from the research**

**Outputs used by…**

**Research Impact**

**(Benefit)**

**Example of application of FAIT Program Logic Model to *Intervention to reduce salt intake, Pacific***

ISSUE

**Aims and activities of the research**

**OUTPUTS from the research**

**OUTPUTS USED BY…**

**IMPACT (OUTCOMES)**
